# Synthesis and Characterization of Hybrid Bio-Adsorbents for the Biosorption of Chromium Ions from Aqueous Solutions

**DOI:** 10.3390/polym18010120

**Published:** 2025-12-31

**Authors:** Nomthandazo Precious Sibiya-Dlomo, Sakhile Cebekhulu, Thembisile Patience Monama, Sudesh Rathilal

**Affiliations:** 1 Green Engineering Research Group, Department of Chemical Engineering, Faculty of Engineering and The Built Environment, Durban University of Technology, Durban 4001, South Africa; cebekhulusakhile0208@gmail.com (S.C.); rathilals@dut.ac.za (S.R.); 2 Postgraduate School of Engineering Management, Faculty of Engineering & the Built Environment, University of Johannesburg, Auckland Park, Johannesburg 2092, South Africa; monamat@uj.ac.za

**Keywords:** linear regression, kinetics, co-precipitation, chromium ions, hybrid-adsorbents

## Abstract

Industrial effluents include toxic chemicals, particularly heavy metals, that remain in the environment and jeopardize human and ecological health. This research synthesized hybrid biosorbents (HBs) for the extraction of Cr (III) from wastewater by using sugarcane bagasse, banana peels, and orange peels in conjunction with magnetite at ratios of 1:2, 1:1, and 2:1. The synthesized biosorbents—MSC, MBP, and MOP—were characterized using FTIR, XRD, TEM, BET, and SEM/EDX, therefore validating their structural, functional, morphological attributes and elementary composition. Batch studies showed MBP (1:1) to be the most efficient sorbent, with over 80% removal of Cr (III). Optimization experiments indicated that the peak removal efficiency (92.10%) was achieved at an initial concentration of 100 mg/L, a pH of 3, a dose of 0.4 g/100 mL, and a contact duration of 60 min. Isotherm analysis revealed that adsorption adhered to a homogeneous monolayer mechanism, optimally characterized by the Langmuir Type 1 model (R^2^ = 0.9688), whereas kinetic analysis demonstrated that the pseudo-second-order model (R^2^ = 0.9419) yielded the most accurate fit. MBP (1:1) has significant promise as an economical and sustainable biosorbent for the efficient removal of Cr (III) from wastewater.

## 1. Introduction

The pollution of water supplies by heavy metal waste has become a significant worldwide environmental and public health concern. Heavy metals—metals and semi-metals exhibiting metallic properties—are well-known for their persistence, toxicity, and possible ecotoxicological impacts [1,2]. Elements including cadmium (Cd), chromium (Cr), copper (Cu), lead (Pb), mercury (Hg), nickel (Ni), selenium (Se), molybdenum (Mo), zinc (Zn), thallium (Tl), and antimony (Sb) are particularly concerning due to their common occurrence in industrial emissions and their established effects on human and ecological health [3,4]. Significant anthropogenic sources include mining, metallurgical and chemical processing, tanneries, battery production, and fossil fuel burning [3,5].

Chromium (Cr) is considered one of the most toxic heavy metals to soil, water, and human health. Chromium ranks as the eighth most prevalent element in the Earth’s crust, with South Africa, Turkey, China, Kazakhstan, and India being the primary worldwide users [6,7,8]. About 84% of the world’s chromite resources are situated in South Africa, with Zimbabwe holding 6%, Kazakhstan 5%, and India 2%; the remainder (3%) is found in Brazil, Russia, Finland, the United States, and Canada [7,9]. Chromium contamination mostly originates from electroplating and metal finishing processes, sewage discharge, and wastewater treatment plants [10]. Moreover, over 90% of leather processing depends on chromium-based tanning, producing extremely hazardous effluents that need rigorous treatment [11,12].

In aquatic environments, chromium mostly occurs as trivalent Cr (III) and hexavalent Cr (VI) [8,10,12]. Cr (III) has poor solubility and restricted mobility, but Cr (VI) is highly soluble, bioavailable, and able to permeate groundwater, hence presenting significant carcinogenic and mutagenic hazards [8,10,12,13]. While Cr (III) is vital for glucose, cholesterol, and lipid metabolism in animals and contributes to liver function, increased levels may provoke dermatological problems and carcinogenic consequences. Moreover, under certain environmental or microbiological circumstances, Cr (III) may be oxidized to Cr (VI), hence intensifying its environmental risk profile regarding minerals [12,14]. Regulatory authorities establish stringent limits: the US Environmental Protection Agency (EPA) requires Cr (VI) concentrations to be below 0.05 mg/L and total chromium below 2 mg/L, while drinking water regulations cap chromium at 0.1 mg/L [8,12]. SANS 241 in South Africa establishes the maximum permissible concentration of total chromium at 50 µg/L [15].

A range of techniques, such as chemical precipitation, ion exchange, coagulation, electrolysis, reverse osmosis, and adsorption, are used to eliminate heavy metals from industrial effluents [10,16,17]. Numerous solutions, nonetheless, are constrained by elevated operating expenses, intricate infrastructure requirements, and substantial sludge production [14,18]. Biosorption has thus garnered heightened interest as a cost-effective, ecological, and efficient alternative. Agricultural residue-derived biosorbents are cost-effective, adaptable, resilient to environmental variations, proficient in recovering important metals, and suitable for continuous treatment systems [10,16,17,19].

Numerous studies have shown the efficacy of iron oxide nanoparticles—specifically maghemite (γ-Fe_2_O_3_), magnetite (Fe_3_O_4_), and haematite (α-Fe_2_O_3_)—in the extraction of heavy metals and hazardous substances from wastewater [3,18,20,21,22]. Agricultural biomasses, including banana peels (BPs), orange peels (OPs), and sugarcane bagasse (SC), have shown efficacy [14,23,24,25] owing to their substantial cellulose, hemicellulose, lignin, and protein content, which offers several functional groups for metal binding [26,27,28,29].

This research examines hybrid bio-adsorbents made from SC, BP, and OP integrated with ferro-magnetite (M) for the removal of Cr (III) from industrial wastewater. The aim is to assess the efficacy of these hybrid materials as economical and sustainable options for industrial wastewater treatment. A thorough characterization was performed utilizing Fourier-transform infrared (FTIR) spectroscopy, Brunauer–Emmett–Teller (BET) surface analysis, X-ray diffraction (XRD), and scanning electron microscopy (SEM) combined with energy-dispersive X-ray (EDX) spectroscopy to evaluate functional groups, surface area, crystalline structure, morphology, and elemental composition. This research promotes Sustainable Development Goal (SDG) 6: Clean Water and Sanitation, and SDG 12: Responsible Consumption and Production, from the 17 United Nations Sustainable Development Goals [30].

## 2. Materials and Methods

### 2.1. Reagents

The chemicals required to prepare stock solutions were analytically graded and supplied by Sigma-Aldrich (Kempton Park, South Africa), including sodium chloride (NaCl), sodium hydroxide (NaOH), hydrochloric acid (HCl, 32%), ferrous sulphate heptahydrate (FeSO_4_·7H_2_O), ferric chloride hexahydrate (FeCl_3_·6H_2_O), oleic acid, chromium nitrate (Cr (NO_3_)_3_·6H_2_O) and 99% ethanol. The stock solutions were prepared using deionized water (ELGA WATERLAB, (High Wycombe, UK)).

### 2.2. Preparation of Synthetic Wastewater Samples

In a volumetric vessel, a synthetic Cr (III) ion solution (1000 mg/L) was prepared by combining 6.66 g of Cr (NO_3_)_3_·6H_2_O with 1 L of distilled water. The desired concentration was achieved by diluting the stock solution with deionized water. Equipped with a portable pH electrode and an integrated temperature sensor that were calibrated with a standard buffer solution, the solution pH was determined using a portable pH meter (Edge pH HI 2002, (Woonsocket, RL, USA)). The pH of the solution was adjusted through the addition of drops of 0.1 M HCl or 0.1 M NaOH.

### 2.3. Preparation of Adsorbents

Bananas and oranges have been purchased from the early morning market in Durban, South Africa. Sugarcane bagasse (SC) was obtained from a local sugar factory in Durban, South Africa. The banana peels (BPs), orange peels (OPs), and SC were rinsed several times to get rid of dirt and impurities. After cleaning, they were dried in the sunlight for 5 days. Subsequently, banana peels (BPs) were oven dried at 120 °C for 12 h [31], while OPs and SC were dried at 105 °C for 24 h [32,33].

Samples were subsequently crushed into a fine powder, sieved through an 850 μm sieve, and stored in an airtight container. Iron oxide nanoparticles (MF) were synthesized via a co-precipitation method using a molar ratio of FeSO_4_·7H_2_O:FeCl_3_·6H_2_O of molar ratio of 1:2, as established in our prior research [34]. Hybrid bio-adsorbents (HBs) were synthesized by combining SC, BP, or OP with MF in three distinct weight ratios: 1:2, 1:1, and 2:1 (Table 1), each weighing 50 g in 500 mL of deionised water, homogenized for 1 h [18]. Following decantation, the precipitates derived from SC, BP, and OP were subjected to oven drying at 105 °C, 120 °C, and 105 °C, respectively, for a duration of 4 h and subsequently stored for characterization.

### 2.4. Characterization

HBs can be analyzed using various techniques to assess their physicochemical properties, including chemical composition, phase purity, size, and shape.

#### 2.4.1. Transmission Electron Microscopy (TEM)

The HBs were dispersed in 100% ethanol and sonicated for 20 min. Thereafter, a carbon-coated Formvar Transmission Electron Microscope (TEM) grid was dipped into the solution, and samples were allowed to air dry. Their images were captured on the High-Resolution Transmission Electron Microscope (JEOL 2100 (Mitaka, Japan)).

#### 2.4.2. X-Ray Diffraction (XRD)

At 40 kV and a target current of 15 mA, the crystal structures of the produced HBs were examined using an X-ray diffractometer (Rigaku MiniFlex600, Tokyo, Japan) that features a sealed ceramic X-ray tube with a copper anode, integrated with Smart Lab software (Studio II v4.5.421.0). The measurements were carried out within the range of 5 to 80° (2*θ*), utilizing a scanning speed of 20°/min and a step width of 0.03°. The data (2*θ* vs. intensity) was graphed utilizing Origin (OriginPro 2019b, 64-bit). The average crystal size of the hybrid bio-adsorbents was determined through the application of the Scherrer equation [35].

#### 2.4.3. Fourier-Transform Infrared (FTIR) Spectroscopy

A Fourier Transform Infrared spectrometer (Shimadzu FTIR 8400) was employed to analyze the organic, polymeric, and inorganic molecular structures and functional groups of the hybrid biosorbents between the 4000–400 cm^−1^ range at a resolution of 5 cm^−1^.

#### 2.4.4. Scanning Electron Microscope (SEM)

The surface morphology and elemental composition of the HBs were analyzed using scanning electron microscopy and energy-dispersive X-ray spectroscopy (SEM/EDX) with a Zeiss Ultra Plus Field Emission Gun Scanning Electron Microscope (FEG SEM), integrated with Oxford INCA and Aztec EDX Analysis Software at the University of KwaZulu, Westville campus, South Africa. This was accomplished using a 20 kV accelerator voltage and magnification of 20 K×.

#### 2.4.5. Brunauer–Emmett–Teller (BET)

The Brunauer–Emmett–Teller (BET) analysis was carried out using Micromeritics TriStar II Plus equipment (Durban, South Africa) and Tristar Plus software version 3.01. Helium and nitrogen were employed as the carrier gases. HBs were weighed (0.5 g) and put into a sample holder on the analyser and degassed separately for 24 h at 150 °C. They were then allowed to cool before being stored under nitrogen gas at a pressure of 5 mmHg for 24 h.

### 2.5. Adsorption Experiments

All adsorption studies were conducted in triplicate at room temperature (25 ± 5 °C) using a batch method. However, average values were employed in the data analysis. The experiments were conducted in a 250 mL conical flask containing 100 mL of chromium solution and homogenized using a temperature-controlled shaker (Scientific Model 262, Scientific Engineering (Pty) Ltd. (Roodepoort, South Africa)) with agitation at 120 rpm [32,36]. Initially, adsorption tests were conducted at pH 5 for the stock solution and pH 3, with an adsorbent dosage of 0.2 g, 30 min contact time, and an initial Cr (III) concentration of 100 mg/L. Thereafter, the best-performing HB was further investigated for the effect of pH (2–6), contact period (30–150 min), starting concentration of Cr (III) (50–250 mg/L), and adsorbent dosage (0.2–1 g). Subsequent to the adsorption procedure, the final product was filtered using Whatman filter paper (150 mm) and PFPE syringe filters (0.45 µm). The collected samples were subsequently analyzed with a plasma atomic emission spectrophotometer (ICPE-9820, Shimadzu, Kyoto, Japan) to quantify the concentration of chromium ions present. The expected elimination percentage (Z (%)) was determined using Equation (1):

(1) $$Z\left(\%\right)=\frac{C_{A}-C_{e}}{C_{A}}\times 100$$

where C_A_ and C_e_ are the initial and equilibrium concentration of Cr (III) in the solution (mg/L), respectively; Z is the percentage removal of HBs (%). The quantity of chromium (III) on HBs will be estimated using the mass balance Equations (2) and (3).

(2) $$Q_{e}=\frac{C_{A}-C_{e}}{M}\times V$$

(3) $$Q_{t}=\frac{C_{A}-C_{t}}{M}\times V$$

where $Q_{e}$ and $Q_{t}$ are amount of the adsorbed chromium ions at equilibrium (mg/g) and at set time (t) in min, respectively; M is the mass of HBs in g; and V is the volume of the adsorbate in L.

### 2.6. Adsorption Kinetics

The modelling of adsorption kinetics facilitated the examination of the biosorption rate over time [16,17]. Kinetic studies are important for understanding the stages that dictate the rate of the adsorption process, as well as for elucidating the process model and the nature of the bond formed between MBP (1:1) and Cr (III). The adsorption process may rely on several mechanisms, including particle diffusion, mass transfer, or chemical reactions [37]. The pseudo-first-order kinetic model, pseudo-second-order kinetic model, Elovich kinetic model, and intra-particle diffusion model were employed to elucidate the kinetic mechanism of the adsorption process. The Lagergren kinetic model, which typically represents first-order adsorption kinetics (Equation (4)), indicates that the rate of loaded adsorbed sites is proportional to the rate of vacant sites [38]. The linear representation of the pseudo-first-order equation is articulated in Equation (5) [39].

(4) $$Q_{t}=Q_{e}\left(1-e^{{-k}_{1}t}\right)$$

(5) $$\log\left(Q_{e}-Q_{t}\right)=\log Q_{e}-\frac{k_{1}t}{2.303}$$

$k_{1}$ is the Lagergren kinetic model rate constant (min^−1^). $Q_{e}$ and $Q_{t}$ are the adsorption capacities at equilibrium (mg/g) and time t (min), respectively. A linear graph of $\log\left(Q_{e}-Q_{t}\right)$ against time (t) yields the values of $k_{1}$ and $Q_{e}$ from the slope and intercept, respectfully. The legitimacy of the adsorption mechanism relies on the regression coefficient values, R^2^, and the projected $Q_{e}$ values acquired [37,39]. The pseudo-second-order kinetic model (Equation (6)), another empirical equation for characterizing adsorption kinetics, and its linear model are defined in Equation (7) [39].

(6) $$Q_{t}=\frac{t}{\frac{1}{K_{2}{Q_{e}}^{2}}+\frac{t}{Q_{e}}}$$

(7) $$\frac{t}{Q_{t}}=\frac{1}{K_{2}{Q_{e}}^{2}}-\frac{1}{Q_{e}}t$$

In this context, $k_{2}$ represents the pseudo-second-order rate constant (g/mg/min). The second-order rate constant ($k_{2}$) is determined from the linearized t versus (t/qt) graph derived from Equation (7). The Elovich kinetic model equations (Equations (8) and (9)) are another kinetic model employed in the study of adsorption kinetic data.

(8) $$\frac{dQ_{t}}{dt}={\alpha e}^{-\beta_{E}Q_{t}}$$

(9) $$Q_{t}=\frac{\ln\left(\alpha \beta_{E}\right)}{\beta_{E}}+\frac{\ln t}{\beta_{E}}$$

In this context, α denotes the initial adsorption rate (mg/g/min), while $\beta_{E}$ signifies the desorption constant (g/mg). By plotting the equation $Q_{t}$ versus lnt (Equation (9)), the Elovich constants can be derived from the resulting line equation [39]. According to Parlayıcı and Baran [38], external mass transfer remains significant even at elevated agitation rates; however, if the boundary layer enveloping the particle in a thoroughly mixed batch system is minimal, or if the agitation rate does not significantly influence the equilibrium, external mass transfer will be markedly diminished. Therefore, the intraparticle diffusion may be the rate-determining factor. Eventually, the intraparticle diffusion model (Equation (10)) was applied to examine the influence of diffusion on the adsorption process, in contrast to the three aforementioned models.

(10) $$Q_{t}=k_{D}t^{0.5}+C$$

where $k_{D}$ signifies the intraparticle diffusion rate constant (g/mg/min^0.5^), and C represents the intercept. A linear graph of $Q_{t}$ versus $t^{0.5}$ is straight and contacts the origin, yielding a slope that indicates $k_{D}$, while C denotes the intercept value, indicating the thickness of the diffusion layer.

### 2.7. Adsorption Isotherms

The quantity of Cr (III) adsorbed per unit mass of MBP (1:1) surface is dependent on C_e_, and numerous adsorption models have been created to represent this relationship. The critical information for a proper understanding of adsorption is adsorption equilibrium data [16]. The Langmuir isotherm more effectively elucidates whether retention in active adsorption sites on solid surfaces is due to physical or chemical adsorption compared to other isotherms [38]. Furthermore, its relation posits that the energy of the adsorbent surface is uniform. Atoms or molecules are held by active centres on the MBP (1:1) surface, and the resulting film is deemed monomolecular. Linear analysis is a superior approach for fitting isotherm data in comparison to nonlinear approaches [35,40,41,42,43]. This study employed the four types of Langmuir, Freundlich, Redlich–Peterson, Dubinin–Radushkevich, and Temkin isotherms. The linear relationships of the five models are presented in Equations (12)–(15), (18), (20), (22), and (25), respectively.

#### 2.7.1. Langmuir Model

The non-linear mathematical form of Langmuir Equation (11) can be linearized into four different types in Equations (12)–(15).

(11) $$Q_{e}=Q_{max}\frac{K_{L}C_{e}}{1+K_{L}C_{e}}$$

(12) $$\frac{C_{e}}{Q_{e}}=\frac{C_{e}}{Q_{max}}+\frac{1}{{K_{L}Q}_{max}}$$

(13) $$\frac{1}{Q_{e}}=\frac{1}{{K_{L}Q}_{max}C_{e}}+\frac{1}{Q_{max}}$$

(14) $$Q_{e}=Q_{max}-\frac{1}{K_{L}}\times \frac{Q_{e}}{C_{e}}$$

(15) $$\frac{Q_{e}}{C_{e}}={K_{L}Q}_{max}-{K_{L}Q}_{e}$$

(16) $$R_{L}=\frac{1}{1+{K_{L}C}_{A}}$$

The graphical representations for types 1 (Equation (12)), 2 (Equation (13)), 3 (Equation (14)), and 4 (Equation (15)) are shown as $\frac{C_{e}}{Q_{e}}$ vs. $C_{e}$, $\frac{1}{Q_{e}}$ vs. $\frac{1}{C_{e}}$, $Q_{e}$ vs. $\frac{Q_{e}}{C_{e}}$, and $\frac{Q_{e}}{C_{e}}$ vs. $Q_{e}$, respectively. Here, $Q_{max}$ (mg/g) is the adsorption capacity and $K_{L}$(L/mg) z is the Langmuir constant related to the energy of adsorption. To assess the suitability of adsorption, the dimensionless $R_{L}$ dispersion constant is calculated, with values between 0 and 1 indicating that the suitability criteria are met. The $R_{L}$ value can be derived using Equation (16). The value of $R_{L}$ indicates whether the bio-sorption process is beneficial (0 < $R_{L}$ < 1), linear (*R_L_* = 1), unfavourable ($R_{L}$ > 1), or irreversible ($R_{L}$ = 0) [29,44,45].

#### 2.7.2. Freundlich Model

The Freundlich isotherm posits that the quantity of Cr (III) adsorbed by a given amount of MBP (1:1) will first rise swiftly, thereafter decelerating as the MBP (1:1) surface approaches saturation. The Freundlich isotherm describes adsorption equilibrium by Equations (17) and (18).

(17) $$Q_{e}=K_{F}C_{e}^{\frac{1}{n_{F}}}$$

(18) $$\log Q_{e}=\log K_{F}+\frac{1}{n_{F}}\log C_{e}$$

$K_{F}$ represents the constants of the Freundlich isotherm, which indicates the intensity of multilayer biosorption, with 1/n representing the biosorption intensity. The phrase 1/$n_{F}$ signifies acceptability when 0.1 < 1/$n_{F}$ < 1 [42]. The plot of $\log Q_{e}$ versus $\log C_{e}$ yields a linear graph with a slope of 1/n. Thus, it facilitates the determination of $K_{F}$ and $n_{F}$.

#### 2.7.3. Redlich–Peterson (R-P) Model

The Redlich–Peterson equations are articulated in Equations (19) and (20), incorporating characteristics of both the Langmuir and Freundlich isotherm assumptions.

(19) $$Q_{e}=\frac{K_{RP}C_{e}}{1+\alpha_{RP}C_{e}^{\beta_{RP}}}$$

(20) $$\ln\left(\frac{C_{e}}{Q_{e}}\right)=\beta_{RP}\ln C_{e}-\ln K_{RP}$$

where $K_{RP}$ (L/mg), and $\alpha_{RP}$ (L/mg) are the Redlich–Peterson constants, and $\beta_{RP}$ is the dimensionless exponent of the Redlich–Peterson model, and it ranges from 0 to 1 due to its two limiting behaviours: the Henry’s law form at $\beta_{RP}$ equal to 0 and the Langmuir form at $\beta_{RP}$ equal to 1 [46,47,48]. At low concentrations, the model approximates Henry’s law, but at large concentrations, it resembles the Freundlich model [39,48,49].

#### 2.7.4. Dubinin–Radushkevich (D-R) Model

This isotherm is predicated on the theory of potential variation on a heterogeneous surface. It serves as an alternative to the Freundlich adsorption isotherm. In the analysis of the D-R isotherm, Equations (21) and (22) were employed, and their expression relies on the adsorption mechanism in compliance with the Gaussian energy distribution [43]. Equation (23) delineates the Polanyi potential (J/mol) as specified in the prior work [49,50,51].

(21) $$Q_{e}=Q_{max}e^{-K_{DR}\varepsilon^{2}}$$

(22) $$\ln Q_{e}=\ln Q_{\max}-K_{DR}\varepsilon^{2}$$

(23) $$\varepsilon =RT\ln\left(1+\frac{1}{C_{e}}\right)$$

where ε (J/mol) represents the Polanyi potential and $K_{DR}$ represents the D-R constant. Also, R and T represent the universal gas constant (8.314 J/mol K) and thermodynamic temperature (K) in this study (298.15 k), respectively.

#### 2.7.5. Temkin Model

Equations (24) and (25) provide the Temkin equations that clarify the influence of indirect interactions between the adsorbent (MBP 1:1) and the adsorbate (Cr (III)) throughout the adsorption process [43].

(24) $$q_{e}=B_{T}lnK_{T}C_{e}$$

(25) $$Q_{e}=B_{T}\ln\left(K_{T}\right){+B}_{T}\ln\left(C_{e}\right)$$

(26) $$B_{T}=\frac{RT}{b}$$

Their characteristics are b (J/mol) and $K_{T}$ (L/mg), which denote the Temkin constant associated with the heat of sorption and Temkin’s equilibrium binding constant, reflecting maximal binding energy, respectively [39,43,49,51,52].

## 3. Results

### 3.1. Characterization

#### 3.1.1. Transmission Electron Microscopy (TEM) Analysis

**Figure 1:**
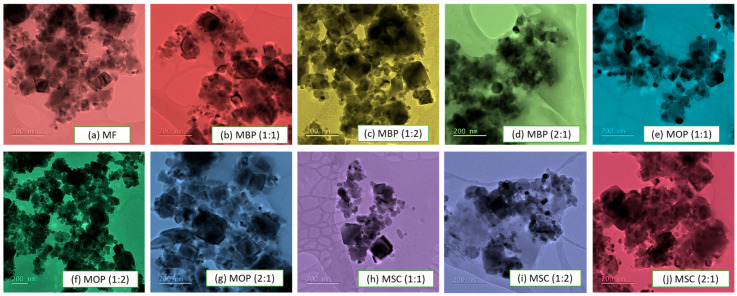
TEM images for the studied HBs.

The morphology of the HBs was studied using TEM (Figure 1). The TEM images of magnetite (Figure 1a) and hybrid bio-adsorbents derived from banana peels (Figure 1b–d), orange peels (Figure 1e–g), and sugarcane bagasse (Figure 1h–j) are displayed. The images were acquired using an X-ray generator operating at 40 kV and 15 mA.

These HBs distinctly exhibit various quasi-spherical morphologies spanning from 0.24 to 0.49 nm, in addition to the appearance of elongated fibrillary forms (Figure S1). The inclusion of MF is evident in all the HBs, where darker particles are observed on the TEM micrographs. Thus, the incorporation of MF was successful.

#### 3.1.2. X-Ray Diffraction (XRD) Analysis

**Figure 2:**
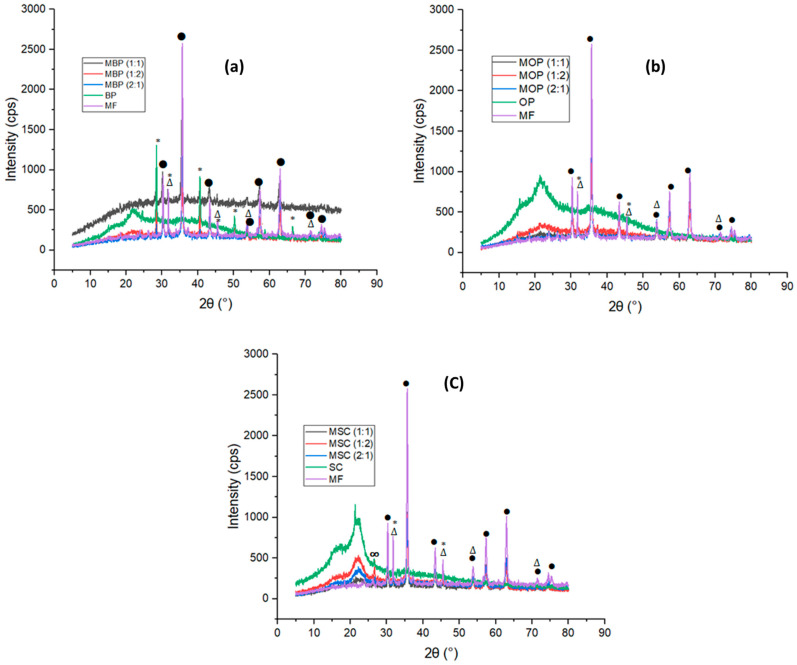
XRD patterns of hybrid bio-adsorbents derived from (**a**) banana peels; (**b**) orange peels; and (**c**) sugarcane bagasse.

XRD investigation was conducted to ascertain the structure, crystallinity, and formation of a metal oxide in HBs [18]. Figure 2 and Table S1 illustrate the XRD patterns and quantitative analysis of each hybrid bio-adsorbent examined, respectively. Figure 2 illustrates X-ray diffraction (XRD) peaks at 30.18°, 35.53°, 43.17°, 53.62°, 57.12°, 62.83°, 74.97°, and 79.42° (2*θ*), which align with the face-centred cubic spinel structure of MF [20,53,54]. The peaks were also observed in the HBs, which confirms the incorporation of MF into the HBs structures. However, in this study, there are additional peaks that indicate potential impurities such as KCl at 28.28° and 40.35°, or mixed phases including NaCl at 31.66°, 45.48°, 53.62°, and 74.97° (Figures S2–S13).

The agricultural waste (OP, BP, and SC) displays wide peaks at 2*θ* = 22°, indicating their mostly amorphous forms [16,33,40,55,56]. This phenomenon may be elucidated by the cleavage of many C-C bonds (the aromatic rings) and the subsequent creation of groups and functionalities on their surfaces [16,35,57,58]. BP (Figure 2a) has pronounced peaks at 24.44°, 28.46°, 40.61°, 50.19°, 58.67°, 66.51°, and 73.88°, indicative of KCl presence. The literature indicates that the prominent diffraction peaks signify crystalline mineral impurities or residual ash content, including silicates, carbonates, and metal oxides [18,56,59,60,61], as seen by peak 26.36 (Figure 2c), which represents graphite. The hybrid bio-adsorbents exhibited similar behaviour with notable peaks at 30.18°, 35,27°, 43.21°, 54.26°,57.61°, 63°, and 74.81°, which correspond to MF. Therefore, the results show the successful synthesis of hybrid bio-adsorbents produced from the co-precipitation method using iron salts with agricultural waste. Hence, Scherer’s equations using a pseudo-Voigt function were used on the peak at 2*θ* of 35.53° to ascertain the dimensions of MF crystallites formed throughout different time intervals [62,63].

#### 3.1.3. FTIR Analysis

**Figure 3:**
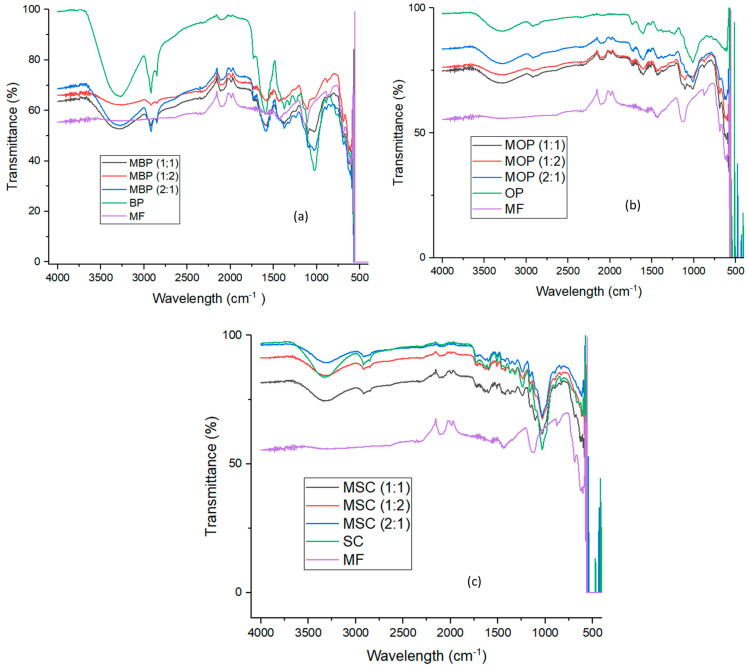
FTIR spectrum of hybrid bio-adsorbents derived from (**a**) banana peels; (**b**) orange peels; and (**c**) sugarcane bagasse.

Figure 3 illustrates the graphs generated by the FTIR analysis of the hybrid bio-adsorbents prior to the adsorption of Cr (III) ions. For each adsorbent, scans were obtained between the range of 4000 to 400 cm^−1^ at a resolution of 5 cm^−1^. The HBs generated from BP, OP, or SC with MF are shown in Figure 3a–c, respectively. The synthesized magnetite (MF) exhibits bands at 2109, 2001, 1461, 1140, 861, 689, 629, and 574 cm^−1^, with large peaks seen at 2109 and 1140 cm^−1^. MF surfaces were coated with hydroxyl groups in an aqueous solution during chemical co-precipitation synthesis [64], as shown by the minor O-H stretching range of 4000 to 3200 cm^−1^.

Also, this indicates that the oleic acid coating effectively lowers surface water adsorption. The broadness of the peaks at 2109 cm^−1^ and 1140 cm^−1^ indicates a distribution of vibrational states, which could be due to hydrogen bonding (O-H or C-O). The distinct signal at 1461 cm^−1^, corresponding to CH_2_ bending in the alkyl chain of oleic acid, clearly indicates efficient surface functionalization. The peaks at 574 cm^−1^ and 629 cm^−1^ are the most distinctive for magnetite [18,38,65] because they pertain to the stretching vibrations of the Fe-O bonds at octahedral sites. All the HBs contain these two peaks, which indicates the MF core in them; hence, the synthesis was successful.Figure 3a shows that in MBP, the stretching vibration of O-H groups, indicative of high cellulose and hemicellulose content, accounts for the broad band at 3302 cm^−1^, while the presence of aliphatic structures is responsible for the C-H stretching bands at 2918 cm^−1^ and 2870 cm^−1^ [31,66]. The C=C and COOH stretching vibrations have pronounced peaks about 1602 cm^−1^ [66], while the bending and deformation vibrations of CH_2_ and CH_3_ occur between 1371 and 1155 cm^−1^ [67]. Lignin’s presence in the structure reinforces the robust C-O vibration peak at about 1025 cm^−1^ [31,61,66]. Aromatic C–H and C–C bonds are classified as moderate and weak in the range of 905 to 500 cm^−1^. The spectrum characteristics of BP align with published values [31,67], with the distinction being peaks at 611 cm^−1^, 582 cm^−1^, 577 cm^−1^, and 562 cm^−1^ due to Si–O bonding in unwashed BP’s gravel and sand.

Figure 3b shows in MOP, bands representing O-H groups in carbohydrates and lignin were seen at 3462 cm^−1^ [16,68]. The band at 1606 cm^−1^ indicates C=C bond stretching in lignin or benzene rings, indicating polysaccharide removal and lignin retention [68]. The small peaks at 2902 cm^−1^ indicate C-H tension from the –CH_2_ group [16,69,70]. Strong cellulose and hemicellulose peaks at 1010 cm^−1^ (C-O-C and C-O-H vibrations) indicated sugar release from orange peels, whereas the band at 1606 cm^−1^ was associated with aromatic C=C stretching from carbonization [68]. The band at 1736 cm^−1^ (carbonyl C=O stretching) indicates the elimination of pectin ester linkages and acetyl group of hemicellulose, indicating effective migration to sugar-rich medium [68,71].

Figure 3c shows in MSC, the spectra of HBs derived from sugarcane bagasse. The results indicate the O–H stretching and bending vibrations at 3350 cm^−1^ and 12,524 cm^−1^, respectively [72,73]. The absorption peak at 1755 cm^−1^ indicates incomplete breakdown of hemicellulose. The spectral band at 1457 cm^−1^ suggested a substantial presence of cellulose [23,73], whereas the absorption band at 1032 cm^−1^ was attributed to the elastic vibration of the C–OH groups [68,74].

#### 3.1.4. SEM/EDX and Surface Area Analysis

**Figure 4:**
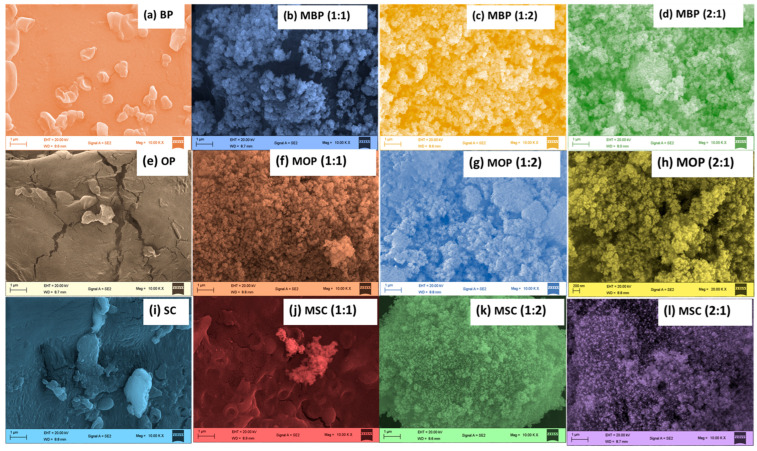
Images SEM of (**a**) BP, (**e**) OP, (**i**) SC, and (**b**–**l**) HBs taken at 1 micrometre scale and 10 K×.magnification.

Figure 4 and Table 2 illustrate the images and chemical compositions of the analyzed HBs and their biomaterials (OP, BP, and SC) before integration with MF. Biomaterials (Figure 4a,e,i) possess uneven and porous surfaces.

The generation of volatile substances such as hydrocarbons, water, carbon monoxide, and carbon dioxide may result in the formation of holes and irregularities [75]. HBs have rough morphologies in cubic shapes, as seen in Figure 4. Furthermore, they demonstrate slight aggregation, accompanied by enhanced pore visibility, consistent with the TEM (Figure 1). EDX results (Table 2) reveal that the primary constituents of the biomaterials are carbon (54.55 to 73.58% C), oxygen (18.89 to 32.78% O), and potassium (2.60 to 11.92% K), with minor quantities of sodium (0.04 to 0.20% Na), silicon (0.10 to 0.76% Si), calcium (0.21 to 1.85% Ca) and phosphorus (0.35 to 0.95% P). The higher concentrations of carbon and oxygen support the concept that the biomaterials are wholly composed of carbohydrate monomers [70,76,77,78]. Potassium plays an important role in adsorption through ion exchange [40,75]. MF exhibited the following composition: Fe (36.48 percent) > O (33.07 percent) > Na (13.8 percent) > S (6.87 percent) > C (9.51 percent) > Cl (0.25 percent). The presence of carbon may result from doping with carbon dioxide during analysis, while other minor elements could improve surface area, facilitating enhanced bio-sorption and reuse [18].

Table 2 demonstrates that HBs contains iron (34 to 66% Fe) > oxygen (22 to 32% O) > carbon (8.36 to 17.58% C) > potassium (0.20 to 6.96% K) > sodium (1.04 to 5.92% Na) > silicon (0.16 to 4.80% Si) > chlorine (0.19 to 3.28% Cl) > sulphur (0.03 to 0.36% S). This verifies that synthesis was successful since Fe and O are prominent [79]. Comparative elemental analysis (Table 2) of biomaterials and HBs reveals a reduction in carbon and trace elements (S, Na, Cl, P, Si, K) alongside an increase in oxygen content. This is consistent with the findings of several previous studies [75,80,81].

The results (Table 2) indicated that the integration of MF into BP, OP, or SC enhanced the surface area of the HBs, thereby augmenting their adsorption capacity, attributed to the unique magnetic properties and stability of MF [20,73,75]. The surface areas of the HBs in descending order are as follows: MBP (2:1) > MBP (1:1) > MOP (2:1) > MBP (1:2) > MOP (1:1) > MOP (1:2) > MSC (2:1) > MSC (1:1) > MSC (1:2).

#### 3.1.5. Point of Zero Charge (PZC)

**Figure 5:**
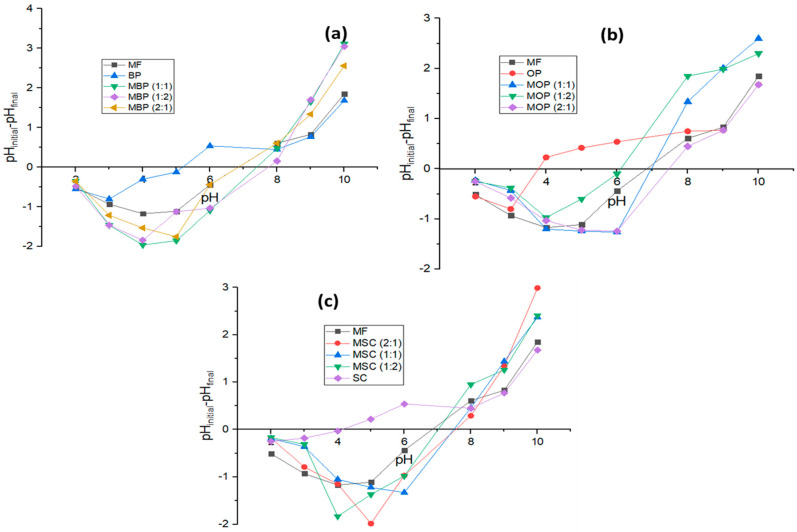
Determination of the point of zero charge for hybrid bio-adsorbents derived from (**a**) banana peels; (**b**) orange peels; and (**c**) sugarcane bagasse.

The point of zero charge (PZC) is regarded as an important component influencing the biosorption capability of the biosorbent and the characteristics of binding sites [33,82]. The method utilized to ascertain the PZC was adapted from Dada et al. [36]. This was accomplished by introducing 0.15 g of HB to 50 mL of 0.1 M NaCl, with the initial pH (pHi) adjusted to values ranging from 2 to 10 with the addition of 0.1 M HCl or 0.1 m NaOH. The final pH (pHf) values were assessed 24 h later, after which the 250 mL conical flasks were enclosed and placed on an orbital shaker (Scientific Model 262, Scientific Engineering (Pty) Ltd.(Roodepoort, South Africa) operating at 150 rpm. The observed difference between pHf and pHi was calculated and displayed against pHi (Figure 5). At the pH corresponding to PZC, the net surface charge of HBs is neutral, resulting in the absence of interaction between the HBs and Cr (III). All HBs exhibit a positive charge at pH levels below the point of zero charge (PZC), which facilitates the adsorption of anionic species, and a negative charge at pH levels above the PZC [42,83].

A recent study has demonstrated that pH 3 is optimal for the removal of Cr (III) in various natural and synthetic adsorbents, including certain lignocellulosic materials [14,37,42]. Therefore, in this study, adsorption experiments were performed at a pH range of 2 to 6 to optimize adsorption. The percentage of ion-exchange decreases rapidly when the pH is increased above 6.0 due to the formation of Cr(III) precipitation and the formation of hydroxyl complexes of chromium, Cr(OH)_3_ at higher pH values The sequence of PZC in decreasing order is as follows MBP (1:2): 7.72 > MBP (1:1): 7.64 > MSC (2:1): 7.39 > MBP (2:1): 7.32 > MSC (1:1): 7.21 > MOP (2:1): 6.86 > MSC (1:2): 6.69 > MOP (1:1): 6.67 > Magnetite: 6.63 > MOP (1:2): 6.06. The ΔpH values exhibited by the HBs below PZC are negative, indicating a preference for accumulating negative charges. The PZC values for orange peels, banana peels, and sugarcane bagasse were determined to be 3.82, 5.25, and 4.05, respectively, which are comparable to those reported in [74,84], [85], [83], respectively. The findings indicate that the point of zero charge for the HBs increased owing to the loading of MF (Figure 5), indicating a greater positive charge on the surface of the HBs.

### 3.2. Effect of Parameters

#### 3.2.1. Performance of Hybrid Bio-Adsorbents

**Figure 6:**
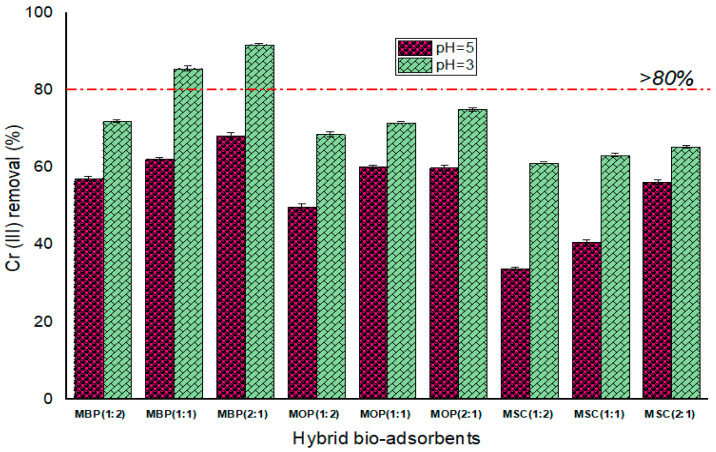
Performance of hybrid bio-adsorbents on Cr (III) removal at room temperature.

Hybrid bio-adsorbents were developed by incorporating MF with either SF, BP, or OP in three proportions: 1:2, 1:1, and 2:1 (Table 1). The findings (Figure 6) reveal that the chromium removal effectiveness improved as the MF ratio rose from 1:1 to 2:1, suggesting that a higher MF concentration may enhance reduction reactivity and alter the surface charge chemistry structure [86]. Nevertheless, augmenting the concentrations of either OP, BP, or SC from 1:1 to 1:2 led to a reduction in Cr (III) removal efficiency, perhaps due to aggregation and obstruction of MF [87]. Zhu et al. [86] proposed that pore aggregation and blockage would impede physisorption and diminish reduction reactivity.

Figure 6 demonstrates that the biosorption of chromium ions is more successful at pH 3, as efficiency improved from pH 5 (23.0% to 68.2%) to pH 3 (63.1% to 91.84%). Similar observations were attained by previous studies that pH 3 is the best for chromium ion removal [10,14,88]. Although the MBP (2:1) ratio appeared optimal (91.84%), MBP (1:1) with 85.61% reduction of chromium ions was employed in subsequent experiments primarily to reduce costs associated with MF. Furthermore, Wang et al. [54] indicated that an excess of nanoparticles could impair the electron transfer efficiency between the carbon matrix and nanoparticles, promote the formation of oxide shells, and consequently diminish reactivity while obstructing the removal of heavy metals.

#### 3.2.2. Effect of pH

**Figure 7:**
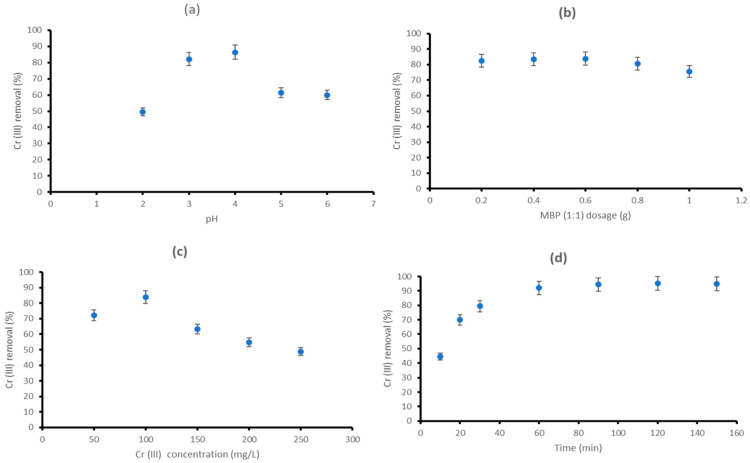
The effect of (**a**) solution pH, (**b**) adsorbent dosage, (**c**) initial chromium concentration, and (**d**) contact time on the removal efficiency for Cr (III) using MBP (1:1).

The pH is also a critical factor influencing the absorption of heavy metal ions from aqueous solutions [16,44,89]. This parameter is closely associated with the competitive capacity of hydrogen ions against metal ions for active sites on the biosorbent surface [52,90]. The influence of initial hydrogen ion concentration on the biosorption of Cr (III) ions onto MBP (1:1) was examined within a pH range of 2 to 6 (Figure 7a). A 100 mL aqueous solution with an initial concentration of 100 mg/L of Cr (III) ions was used with 0.2 g of MBP (1:1) in a 250 mL flask. The solution was stirred at 120 rpm for 30 min at 25 ± 5 °C During the assessment of pH values both the commencement and conclusion of the biosorption process, it was that, regardless of the starting pH (acidic or basic), the equilibrium pH value increased from an initial acidic state and declines from an initial basic state towards neutrality (slightly acidic). At pH (2), the biosorption efficiency of Cr (III) was reduced due to competition between Cr (III) ions and protons for binding to accessible sites. The removal efficiency of Cr (III) ions improved as the pH increased from 2 to 4 (49.5% to 86.4%), indicating that an increase in solution pH enhances the negative charge on the surface of MBP (1:1) and promotes the deprotonation of its functional groups, thereby making them more accessible to Cr (III) ions [42,52]. Comparable pH behaviour has been shown in Cr (III) investigations [14,37,42]. In this study, it was noticed that when pH exceeds 4, the removal effectiveness diminishes, suggesting chromium hydroxide precipitates formed [42,52,91,92]. The research conducted by Lugo-Lugo et al. [14] also showed that Cr (III) ions are predominant until around pH 2, at which point Cr(OH)^2+^ starts to develop. Furthermore, it showed that the concentration of this hydroxide complex increases at the cost of the free cation as the pH rises from 2 to about 4, at which point no free cation remains, and the insoluble hydroxide precipitate Cr (OH)_3_(s) commences formation. As the pH increases from 4 to 6, their observation showed that the quantity of insoluble hydroxide starts to increase, whilst the concentration of the hydroxide complex decreases. They concluded that at pH 6, the precipitate became the predominant species [89,92]. In this study, the optimal removal occurred at around pH 4 but pH of 3 (82.1%) was selected for following studies to enhance adsorption conditions while preventing chromium precipitation. This pH is below the P_ZC_ of 7.64, resulting in a positively charged surface of MBP (1:1). Therefore, the biosorption behaviour of Cr (III) was predicated on the principles of ion exchange and hydrogen bonding [42,83,89].

#### 3.2.3. Effect of MBP (1:1) Dosage

The impact of the MBP (1:1) dosage on the adsorption of Cr (III) was examined. A batch-scale experiment was conducted at room temperature (25 °C) by varying the amount from 0.2 to 1 g, maintaining the solution’s (100 mg/L) pH at 3, and allowing a contact period of 30 min. The results (Figure 7b) showed that Cr (II) removal efficiency escalated from 82.59% to 83.49% when the dose rose from 0.2 g to 0.4 g, and with a further rise to 0.6 g, it marginally rose to 83.85%. This suggests that the biosorption sites are unsaturated throughout the adsorption process; nevertheless, the quantity of available biosorption sites increases with a higher dose of MBP (1:1), attributed to the increased surface area from the addition of a larger amount of MBP (1:1). The findings indicate that when the dosage of MBP (1:1) surpasses 0.6 g, chromium biosorption declines, resulting in a reduction in elimination efficiency owing to the overlapping, aggregation, and stacking effects of MBP (1:1) caused by their elevated concentration levels, which lead to particle aggregation [70,93]. Pradhan et al. [94] indicated that increased Cr-loading at reduced MBP (1:1) dose is advantageous for industrial applications. Hence, 0.4 g was chosen for further experiments.

#### 3.2.4. Effect of Initial Cr (III) Concentration

The initial concentration of Cr(III) as an adsorbate significantly influences the biosorption capacity, since biosorption is a physical process involving mass diffusion at the interface of two phases [36,94]. This study (Figure 7c) assessed the effect of altering Cr (III) starting concentration (50 to 150 mg/L) at the following experimental conditions of pH 3, contact time 60 min, a dose of 0.4 g for MBP (1:1), and temperature 25 °C. The results show that the Cr (III) biosorption potential of the MBP (1:1) increased with an increase in initial feed concentration. The findings indicated that the removal effectiveness rose from 72.21% to 83.9% when the initial Cr (III) concentration increased from 50 to 100 mg/L (Figure 7c). The concentration gradient facilitating the biosorption process was significantly enhanced by elevated beginning feed concentrations, leading to better Cr (III) reduction. Subsequently, efficiency diminished consistently from 83.9% to 48.81% when the starting Cr (III) concentration escalated from 100 to 250 mg/L. This may result from insufficient active sites in MBP (1:1) for the diffusion of the escalating concentration of Cr (III), or from reciprocal collisions that impede diffusion at the interface of the two phases, or from conflicting repulsive interactions among them [70,94,95]. A feed solution with a starting concentration (100 mg/L) is preferable for MBP (1:1).

#### 3.2.5. Effect of Contact Time

The impact of contact time on the biosorption of Cr (III) by MBP (1:1) was estimated by studying the biosorption at different intervals of time (10 min,20 min, 30 min, 60 min, 90 min, 120 min, and 150 min). The 250 mL of Cr (III) solution, having a 100 mg/L initial concentration and 0.4 g MBP (1:1), was used in a 250 mL flask. The mixture was agitated at 120 rpm for different intervals of time. Figure 7d illustrates the elimination of Cr (III) adsorption using MBP (1:1) over different contact durations (10 to 150 min). The removal efficiency surged dramatically during the first 30 min and thereafter rose at a slower rate until achieving biosorption equilibrium at 60 min (Figure 7d). The removal effectiveness increased from 44.50% to 92.10% when the contact duration was prolonged from 10 to 60 min. This rapid initial increase may be ascribed to the availability of more active adsorption sites for Cr (III) ion binding on the MBP (1:1) surface and the elevated concentration gradient at the onset of adsorption [53]. A similar trend was attained from previous studies [16,33,36,52,70,95]. After 60 min, the active sites were saturated with Cr (III) ions, as shown by the minimal rise in removal efficiency (92.1 to 94.9%) due to the presence of residual active sites.

### 3.3. Adsorption Kinetics

**Figure 8:**
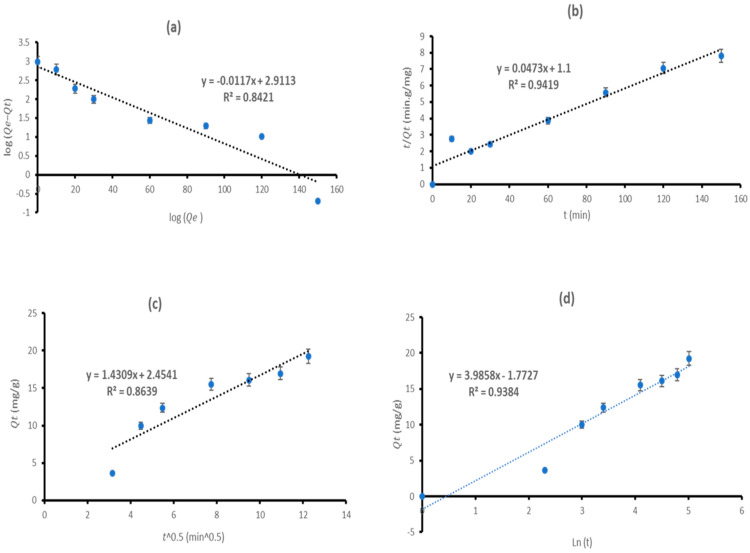
Linearized fitting of pseudo-first-order (**a**), pseudo-second-order (**b**), intra-particle diffusion (**c**), and Elovich (**d**) models for adsorption kinetics of Cr (III) onto MBP (1:1).

Kinetic studies were conducted to examine the relationship between contact time and kinetic adsorption capacity. The operating conditions for this kinetics were met by using 0.4 g MBP (1:1), 100 mg/L Cr (III), and duration (0–150 min) at room temperature. To ascertain the precise characterization of experimental outcomes, linear kinetics, including pseudo-first-order, pseudo-second-order, intra-particle diffusion, and Elovich models, together with their mathematical representations and corresponding parameters in Equations (5, 7, 9, and 10, respectively), were used. Linear regression analysis, specifically the coefficient of determination (R^2^), was used to examine the linear representations of kinetic models (Figure 8 and Table 3). Kinetic constants were ascertained using the slope and intercept values from the linear plots. Although all analyzed kinetic models exhibited an adequate fit (R^2^ > 0.84) to the experimental data, the pseudo-second-order model emerged as the most precise representation (R^2^ = 0.9419) for Cr (III) sorption on MBP (1:1). It is further shown by the equilibrium adsorption capacity values ($Q_{e}$ = 21.1416 mg/g), which closely aligns with the experimental data ($Q_{e,exp}$ = 21.0 mg/g). The pseudo-second-order model posits that the rate-controlling stage of the adsorption process is chemisorption, which entails the sharing or exchange of electrons between adsorbents and metal ions [49,53,95]. Consequently, the adsorption of Cr (III) onto MBP (1:1) was determined to be a chemical process rather than physisorption. Similar observations were found in previous studies (Table 4).

### 3.4. Isotherms

**Figure 9:**
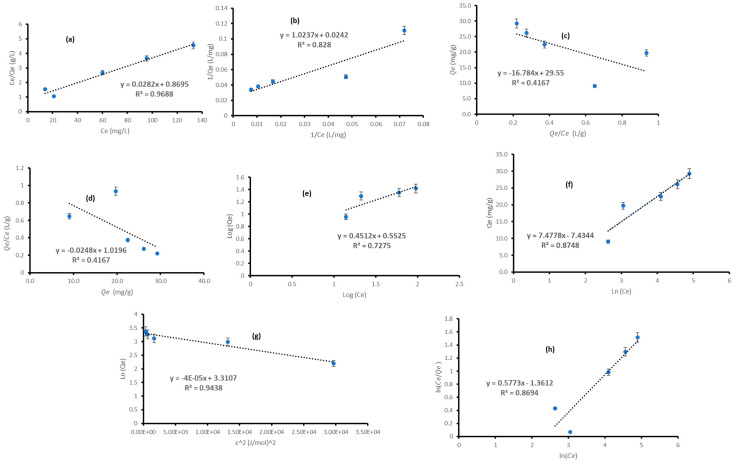
Linearized fitting of Langmuir Type 1 (**a**), Type 2 (**b**), Type 3 (**c**), Type 4 (**d**), Freundlich (**e**), Temkin (**f**), Dubinin–Radushkevich (D-R) (**g**), and Redlich–Peterson (R-P) (**h**) models for adsorption isotherms of Cr (III) on MBP (1:1) 3.5. Comparative studies for removal efficiencies for the removal of Cr (III).All adsorption systems shown in Table 5 conformed to the PSO kinetic model but did not adhere to only the Langmuir isotherm model, despite the removal of a comparable pollutant. Therefore, it is recommended to fit the data to many isotherm models (Table 4) to ascertain that the most accurately fitted model reflects the true adsorption process at equilibrium.

The adsorption isotherm is an important design instrument that delineates the relationship between the adsorbate and adsorbent at equilibrium [92,94,95,96]. The criteria defining it include temperature, pH, and initial concentration of the adsorbate, which reflect the surface properties and the adsorbent’s affinity for metal ion adsorption [39,94]. This enables the researcher to ascertain the kind of adsorption process—whether physical or chemical—and provides insight into the surface characteristics of the adsorbent [39]. This investigation used 0.4 g MBP (1:1), pH 3, and Cr (III) concentrations ranging from 50 to 250 mg/L. The experiments were performed in triplicate, and the average results were reported. The experimental equilibrium data for the adsorption of Cr (III) on MBP (1:1) were fitted to two-parameter (Langmuir, Freundlich, Temkin and D-R) and three-parameter (P-R) isotherm models. All isotherms were presented in their linear versions displayed by Equations (12)–(15): Langmuir; 18: Freundlich; 20: P-R; 22: D-R; and 25: Temkin. Figure 9 illustrates the fitting of experimental data by several models, while Table 4 summarizes the parameters of each model. The examination of the two-parameter adsorption isotherm models revealed that the Langmuir type 1 isotherm effectively characterized the adsorption of Cr (III) ions onto MBP (1:1) from the aqueous solution, as shown by the greatest correlation coefficient (R^2^) of 0.9668. The $R_{L}$ value ranged from 0 to 1, indicating the favorability of the adsorption process under the examined circumstances [94].

The Langmuir monolayer adsorption capacity ($Q_{m}$) was determined to be 34.4566 mg/g, suggesting a superior adsorption capacity of MBP (1:1) in comparison to the experimental value of 21 mg/g. The $K_{L}$ value (0.0325 L/mg) was notably elevated, indicating substantial surface energy in the process and, therefore, strong bonding between Cr (III) ions and MBP (1:1).

The Freundlich isotherm was found to align with the experimental data, with a correlation value of 0.7275. The result of $n_{F}$ (2.2163) > 1 indicated that the adsorption phenomena were physical [42,96]. The ratio of $1/n_{f}$ (0.4512) was less than one, indicating a chemisorption process according to [51].

The correlation coefficient (0.9438) derived from the D-R isotherm plot indicated a strong match of the equilibrium data to the model. The Dubinin–Radushkevich constant ($K_{DR}$) of 4.0 × 10^−5^ yielded a mean sorption energy of 114.7801 J/mol, indicating a physisorption process [97]. The D-R model, predicated on the pore-filling process, yielded a $Q_{m}$ value of 27.4043 mg/g, which is more aligned with the experimental data ($Q_{e,exp}$ = 21 mg/g) than the Langmuir model, despite a lower R^2^.

The experimental findings conformed closely to the Temkin isotherm, exhibiting a high correlation coefficient (R^2^) of 0.8748, which suggests that the adsorption of Cr (III) ions transpired due to the linear reduction in the heat of adsorption of all ions inside the layer as surface coverage increased [51].

The analysis of the three-parameter isotherm model (R-P) indicated that the experimental data exhibited a strong match, with a correlation coefficient of 0.8694 and relatively high values of $\beta_{RP}$ and $K_{RP}$. The R^2^ value derived from type 1 (0.9688) of the linearized Langmuir equation is the greatest in comparison to the other three variants (Type 2:0.8280; Type 3 = 4:0.4167), followed by D-R (0.9438) > Temkin (0.8748) > R-P (0.8694) > Freundlich (0.7275). Based on the results, it was noted that the equilibrium data aligned more closely with Langmuir type 1. As a result, this evidence indicates that the adsorption process of Cr (III) mostly happens via monolayer coverage, which aligns with previous studies in Table 5 [14,37,42,92,98].

**Table 5:** Comparison of the adsorption capacities of Cr (III) with other biosorbents in the literature.

Adsorbent/s	Working Conditions Efficiency	Removal Efficiency	Adsorption Capacity (mg/g)	Best Fitted Isotherm	Kinetics	Reference
MBP (1:1)	0.4 g;100 mg/L; 60 min and pH = 3	92.10%	35.46	Langmuir	PSO	In this study
Sawdust (treated)	1 g; 513.72 mg/L; 120 min and pH = 2.5; 27.5 °C	99.27%	4.69	Langmuir	PSO	[42]
Corn husk (treated)		99.16%	4.70	Langmuir	PSO	
Immobilized corn cob biomass	0.1 g; 100 mg/L, 24 hr and pH = 5; 25 °C	64.52%	277.57	Langmuir	PSO	[92]
Pre-treated orange peel	1 g;10 mg/L; T = 25 °C; 240 min; pH = 3	79%	9.43	Langmuir	PSO	[14]
Magnetic calcite	0.5 g/L;10 g/L; T = 40 °C; 60 min; pH = 6.0	94%	24.2	Langmuir and Freundlich	PSO	[37]
Jackfruit peel	0.4 g;10 g/L; T = 25 °C; 30 min	-	13.50	Langmuir	PSO	[98]
NaOH-modified peel of Artocarpus nobilis fruit	0.2 g, 10 mg/L; 120 min and pH = 5; 25 °C	-	4.87	-	PSO	[71]

## 4. Conclusions

Chromium (III) is toxic and poses a risk to aquatic life and humans. Different ratios of banana peels, orange peels, and sugarcane bagasse were mixed with magnetite to make hybrid bio-adsorbents (HBs). Characterization included SEM/EDX, BET, FTIR, XRD, and TEM. MBP (2:1) removed 91.84% chromium, best in biosorption tests, while MBP (1:1) preserved magnetite best. The optimal conditions (pH 3, 100 mg/L Cr (III), 0.4 g, 60 min) removed 92.10%. Data fit best with the Langmuir isotherm (R^2^ = 0.9688) and pseudo-second-order kinetics (R^2^ = 0.9419). HBs removed chromium and will be tested for copper and lead, with reuse strategies to reduce secondary waste.

## Figures and Tables

**Table 1:** Quantities assessed for agricultural waste and iron oxide nanoparticles to produce 50 g of HBs.

HB Ratio/(s)	MOP		MBP		MSC	
	MF (g)	OP (g)	MF (g)	BP (g)	MF (g)	SC (g)
1:1	25	25	25	25	25	25
1:2	16.67	33.33	16.67	33.33	16.67	33.33
2:1	33.33	16.67	33.33	16.67	33.33	16.67

MOP—magnetized orange peels, MBP—magnetized banana peels, MSC—magnetized sugarcane bagasse.

**Table 2:** The elemental content and surface area of the studied HBs and their biomaterials.

Sample/s	Surface Area (m^2^/g)	C	O	K	S	Ca	Fe	P	Si	Na	Cl
MF	38.1951	9.51 ± 0.57	33.07 ± 0.32	-	6.87 ± 0.11	-	36.48 ± 0.32	-	-	13.80 ± 0.20	0.25 ± 0.04
BP	3.6689	54.54 ± 0.44	32.78 ± 0.39	11.71 ± 0.13	-	0.21 ± 0.05	-	0.53 ± 0.04	0.18 ± 0.03	0.04 ± 0.05	-
OP	2.7196	73.58 ± 0.46	18.89 ± 0.43	4.43 ± 0.09	-	1.85 ± 0.07	-	0.95 ± 0.09	0.10 ± 0.09	0.20 ± 0.09	-
SC	1.8891	65.76 ± 0.37	30.47 ± 0.36	2.20 ± 0.02	-	0.40 ± 0.02-	-	0.35 ± 0.13	0.76 ± 0.03	0.06 ± 0.13-	-
MBP (1:1)	47.5190	17.58 ± 0.46	31.22 ± 0.36	6.95 ± 0.08	-	0.36 ± 0.03	34.01 ± 0.07	-	0.68 ± 0.03	5.92 ± 0.09	3.28 ± 0.05
MBP (1:2)	34.9912	15.78 ± 0.66	22 ± 0.27	1.34 ± 0.05	-	0.02 ± 0.04	57.49 ± 0.07	-	0.17 ± 0.05	2.08 ± 0.12	1.12 ± 0.05
MBP (2:1)	48.1893	8.24 ± 0.77	37.20 ± 0.41	7.46 ± 0.11		0.03 ± 0.15	38.05 ± 0.39	-	4.80 ± 0.09	4.03 ± 0.13	0.19 ± 0.11
MOP (1:1)	31.9438	16.90 ± 0.44	22.34 ± 0.24	9.02 ± 0.39-	-	-	50.15 ± 0.39	-	0.16 ± 0.04	1.04 ± 0.09	0.39 ± 0.04
MOP (1:2)	30.7021	8.36 ± 0.69	31.62 ± 0.33	0.14 ± 0.04	-	-	59.45 ± 0.50	-	0.15 ± 0.04	-	0.28 ± 0.04
MOP (2:1)	39.1918	16.01 ± 0.65	27.42 ± 0.31	0.20 ± 0.04-	-	-	55.36 ± 0.49	-	0.27 ± 0.04	-	0.74 ± 0.04
MSC (1:1)	28.2011	19.28 ± 0.67	28.83 ± 0.40	0.05 ± 0.03-	-	-	51.24 ± 0.40	-	0.35 ± 0.04	-	0.25 ± 0.03
MSC (1:2)	20.8773	13.91 ± 0.46	26.38 ± 0.26	0.03 ± 0.04-	-	-	59.49 ± 0.38	-	-	-	0.19 ± 0.04
MSC (2:1)	30.15291	10.01 ± 0.44	22.49 ± 0.24	0.89 ± 0.10	-	-	65.83 ± 0.39	-	0.29 ± 0.04		0.49 ± 0.04

**Table 3:** Parameters for kinetic models of Cr (III) sorption on MBP (1:1).

Kinetic Model	Coefficients $Q_{e,exp}$ = 21 mg/g	Value
PFO	$Q_{e}$ mgg	819.0304
	K1 1min	0.0269
	$R^{2}$	0.8421
PSO	Qe mgg	21.1416
	K2 gmg.min	0.0020
	$R^{2}$	0.9419
Intra-particle	Qt mgg	16.0288
	KD mgg.min0.5	1.4309
	C	2.4541
	$R^{2}$	0.8639
Elovich	Qt mgg	16.1590
	α gmg.min	2.5541
	βEgmg	0.2509
	$R^{2}$	0.9384

**Table 4:** Linear isotherm parameters and correlation coefficients (R^2^) for the adsorption of Cr (III) over MBP (1:1).

Isotherm Model	Isotherms Parameter/(s)	Type 1	Type 2	Type 3	Type 4
Langmuir	$Q_{m}$ mgg	35.4610	41.3223	29.5500	41.1129
	KL Lmg	0.0325	0.0236	0.0596	0.0248
	$R_{L}$	0.2357	0.2973	0.1437	0.2874
	$R^{2}$	0.9688	0.8280	0.4167	0.4167
Freundlich	KF mg/gL/mg1/n	3.5686			
	$1/n_{F}$	0.4512			
	$n_{F}$	2.2163			
	$R^{2}$	0.7275			
Temkin	$K_{T}$ Lmg	0.3700			
	b mgg	331.4904			
	$R^{2}$	0.8748			
D-R	Qmax mgg	27.4043			
	$K_{DR\;}$	0.00004			
	ε (J/mol)	114.7806			
	$R^{2}$	0.9438			
R-P	KRP mgg	3.9009			
	$\beta_{RP}$	0.5773			
	$R^{2}$	0.8694			

## Data Availability

The original contributions presented in this study are included in the article/Supplementary Material. Further inquiries can be directed to the corresponding author.

## Supplementary Materials

The following supporting information can be downloaded at: https://www.mdpi.com/article/10.3390/polym18010120/s1. Figure S1: Spectra from TEM for magnetite (MF) showing the diameter of particles; Figures S2–S13: Qualitative analysis of HBs; Table S1: Characteristics of the HBs analyzed using X-ray diffraction (XRD).
