# Supplementary material for: Synthesis and Characterization of Hybrid Bio-Adsorbents for the Biosorption of Chromium Ions from Aqueous Solutions
Source: Polymers (Basel). 2025 Dec 31;18(1):120. doi: 10.3390/polym18010120 (PMC12787901; doi:10.3390/polym18010120)
Supplement: Supplementary file 1 [file polymers-18-00120-s001.zip › polymers-3988238-supplementary.pdf]

Synthesis and characterisation of hybrid bio-adsorbents for the biosorption of chromium ions from aqueous solutions

Nomthandazo Precious Sibiya-Dlomo*[1], Sakhile Cebekhulu[1], Thembisile Patience Monama[2], and Sudesh Rathilal[1]

1.Green Engineering Research Group, Department of Chemical Engineering, Faculty of Engineering and The Built Environment, Durban University of Technology, Durban 4001, South Africa

2. Postgraduate School of Engineering Management, Faculty of Engineering & the Built Environment, University of Johannesburg, Auckland Park 2092, South Africa

cebekhulusakhile0208@gmail.com (S.C); monamat@uj.ac.za (T.P.M); rathilals@dut.ac.za (S.R.)

* Correspondence: NomthandazoS3@dut.ac.za (N.P.S.) Tel.: +27-63205-5378 (N.P.S-D.)

Table S1 Characteristics of the HBs analysed using X-ray diffraction (XRD)

Chemical Formula	Phase name	Crystal structure	Card No	Representation symbol
KCl	Sylvite	Face-centered cubic	9003114	*
NaCl	Halite	Face-centered cubic	9006375	∆
Fe_3O_4	Magnetite	Face-centered cubic	9002318	●
C	Graphite	Hexagonal layered	9008569	∞

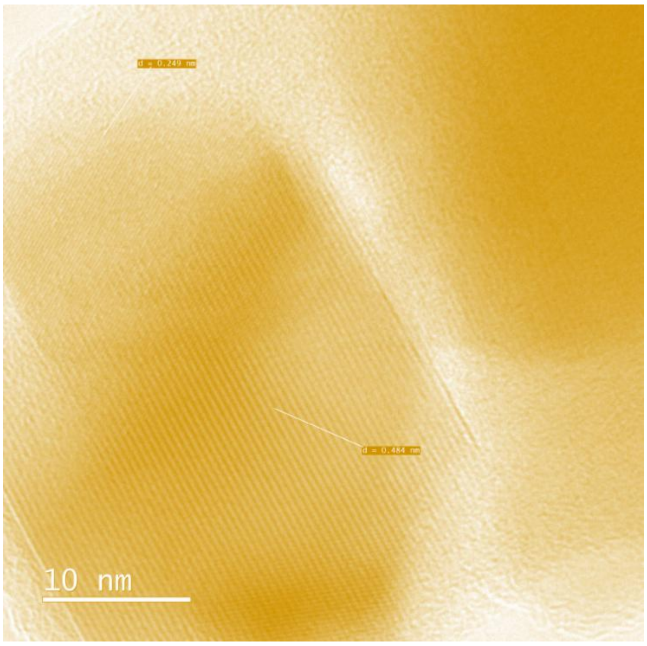

Figure S1 Spectra from TEM for magnetite (MF) showing the diameter of particles

Qualitative Analysis Results

Phase name	Chemical formula	FOM	Phase reg. detail	Space Group	DB Card Number
Sylvite	KCl	0.981	S/M:COD	225 : Fm-3m	9003114

Phase Data View

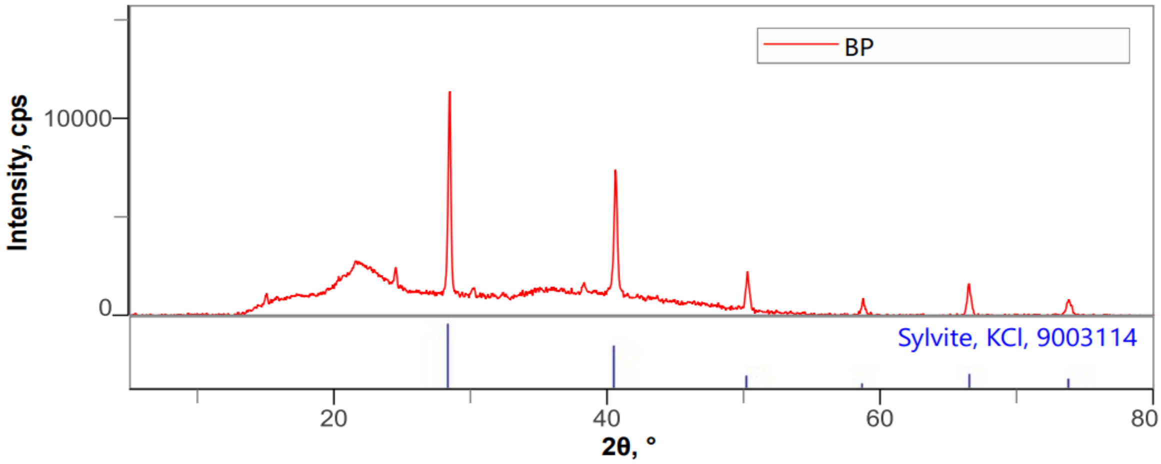

Figure S2 Qualitative analysis of banana peels (BP)

Qualitative Analysis Results

Phase name	Chemical formula	FOM	Phase reg. detail	Space Group	DB Card Number
Magnetite	Fe3 O4	0.243	S/M:COD	227 : Fd-3m:2	9002318
Sylvite	KCl	0.492	S/M:COD	225 : Fm-3m	9003114
Halite	Cl Na	0.617	S/M:COD	225 : Fm-3m	9006375

Phase Data View

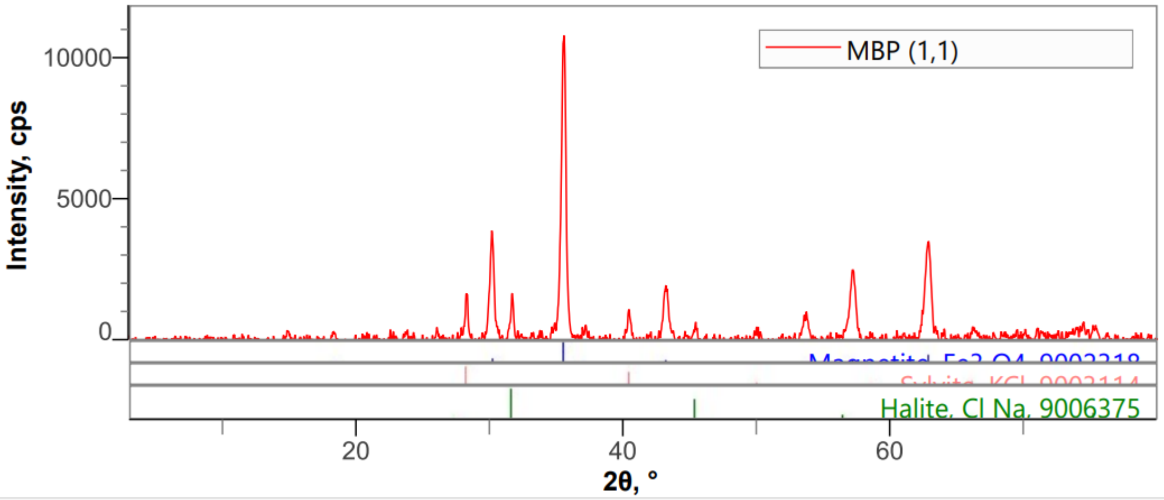

Figure S3 Qualitative analysis of MBP (1:1)

Qualitative Analysis Results

Phase name	Chemical formula	FOM	Phase reg. detail	Space Group	DB Card Number
Sylvite	KCl	0.492	S/M:COD	225 : Fm-3m	9003114
Halite	Cl Na	0.617	S/M:COD	225 : Fm-3m	9006375
Magnetite	Fe3 O4	0.243	S/M:COD	227 : Fd-3m:2	9002318

Phase Data View

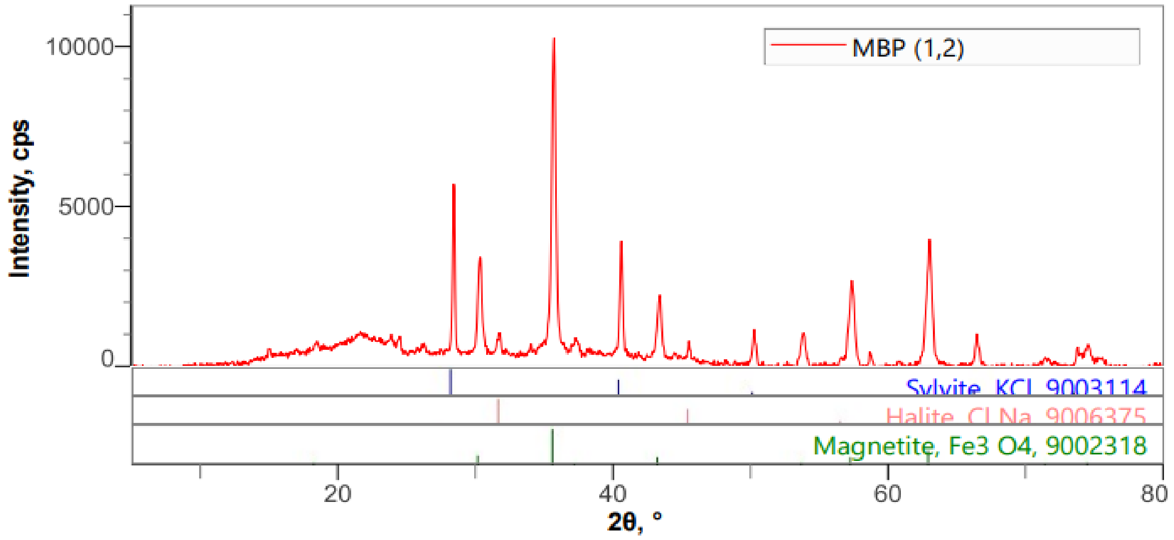

Figure S4 Qualitative analysis of MBP (1:2)

Qualitative Analysis Results

Phase name	Chemical formula	FOM	Phase reg. detail	Space Group	DB Card Number
Halite	Cl Na	0.617	S/M:COD	225 : Fm-3m	9006375
Magnetite	Fe3 O4	0.243	S/M:COD	227 : Fd-3m:2	9002318
Sylvite	KCl	0.492	S/M:COD	225 : Fm-3m	9003114

Phase Data View

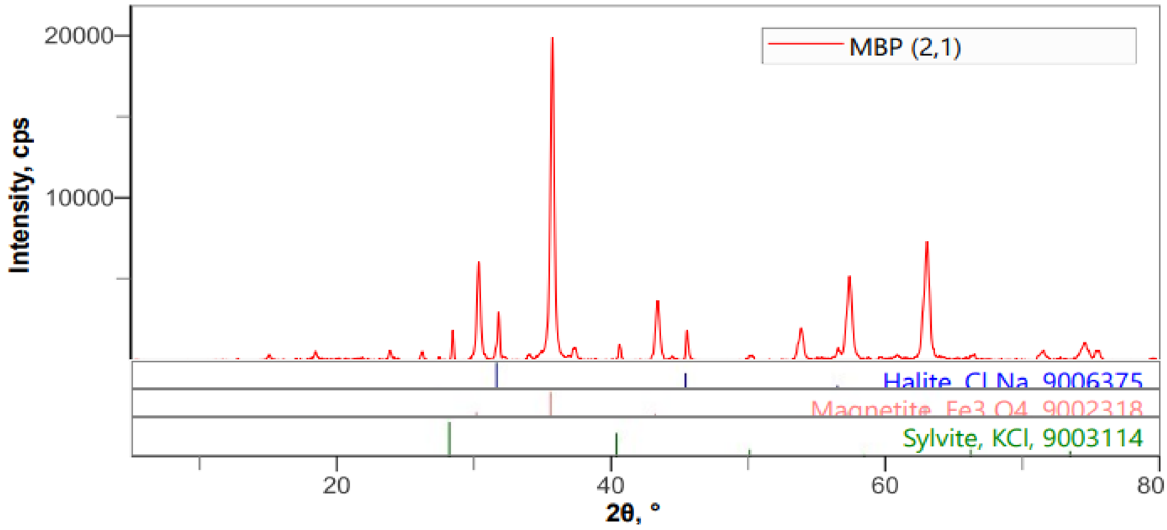

Figure S5 Qualitative analysis of MBP (2:1)

Qualitative Analysis Results

Phase name	Chemical formula	FOM	Phase reg. detail	Space Group	DB Card Number
Magnetite	Fe3 O4	0.243	S/M:COD	227 : Fd-3m:2	9002318
Sylvite	KCl	0.492	S/M:COD	225 : Fm-3m	9003114
Halite	Cl Na	0.617	S/M:COD	225 : Fm-3m	9006375

Phase Data View

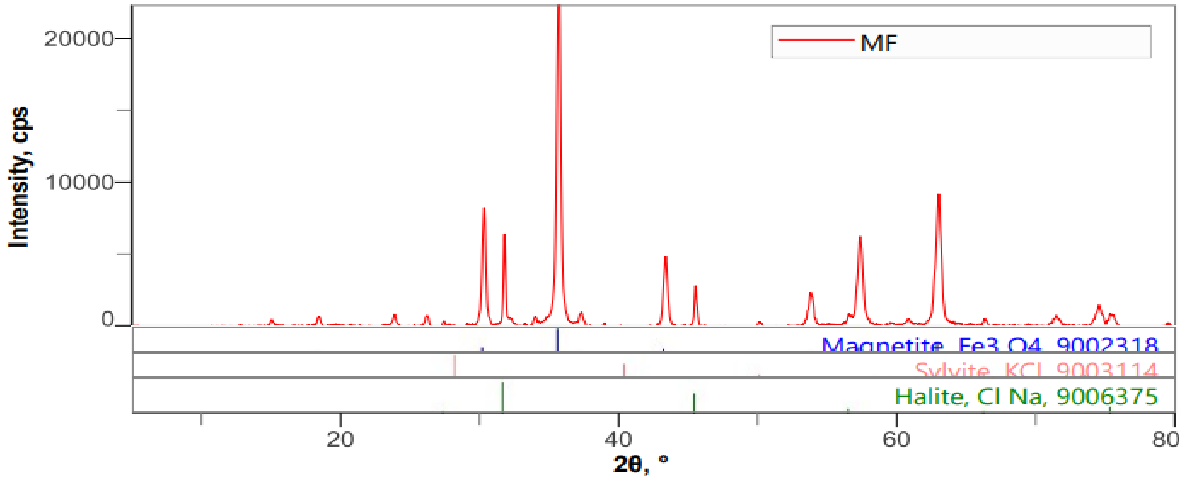

Figure S6 Qualitative analysis of MF

Qualitative Analysis Results

Phase name	Chemical formula	FOM	Phase reg. detail	Space Group	DB Card Number
Halite	Cl Na	1.087	S/M:COD	225 : Fm-3m	9006375
Magnetite	Fe3 O4	0.372	S/M:COD	227 : Fd-3m:2	9002318

Phase Data View

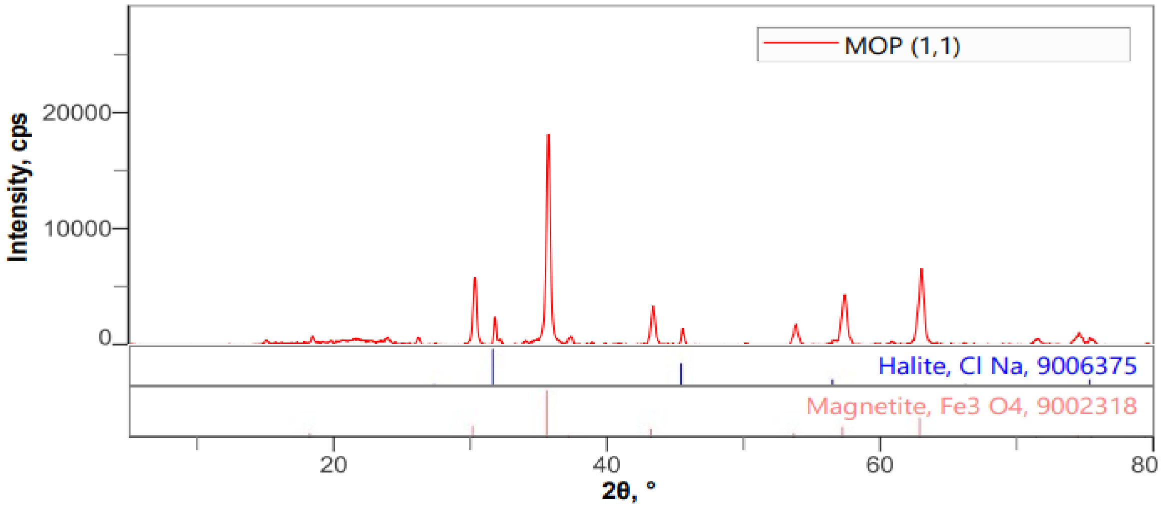

Figure S7 Qualitative analysis of MOP (1:1)

Qualitative Analysis Results

Phase name	Chemical formula	FOM	Phase reg. detail	Space Group	DB Card Number
Halite	Cl Na	1.087	S/M:COD	225 : Fm-3m	9006375
Magnetite	Fe3 O4	0.372	S/M:COD	227 : Fd-3m:2	9002318

Phase Data View

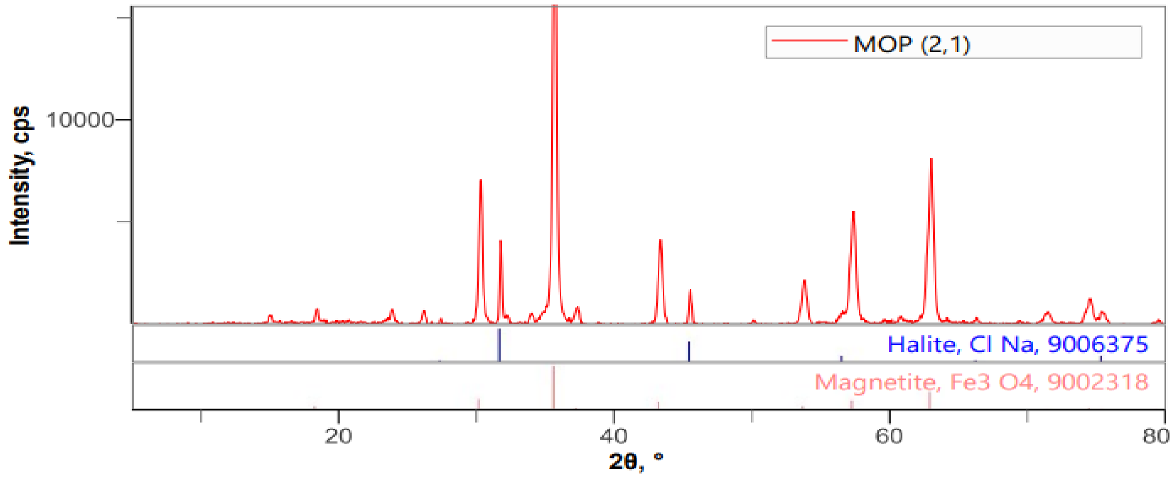

Figure S8 Qualitative analysis of MOP (2:1)

Qualitative Analysis Results

Phase name	Chemical formula	FOM	Phase reg. detail	Space Group	DB Card Number
Magnetite	Fe3 O4	0.372	S/M:COD	227 : Fd-3m:2	9002318
Halite	Cl Na	1.087	S/M:COD	225 : Fm-3m	9006375

Phase Data View

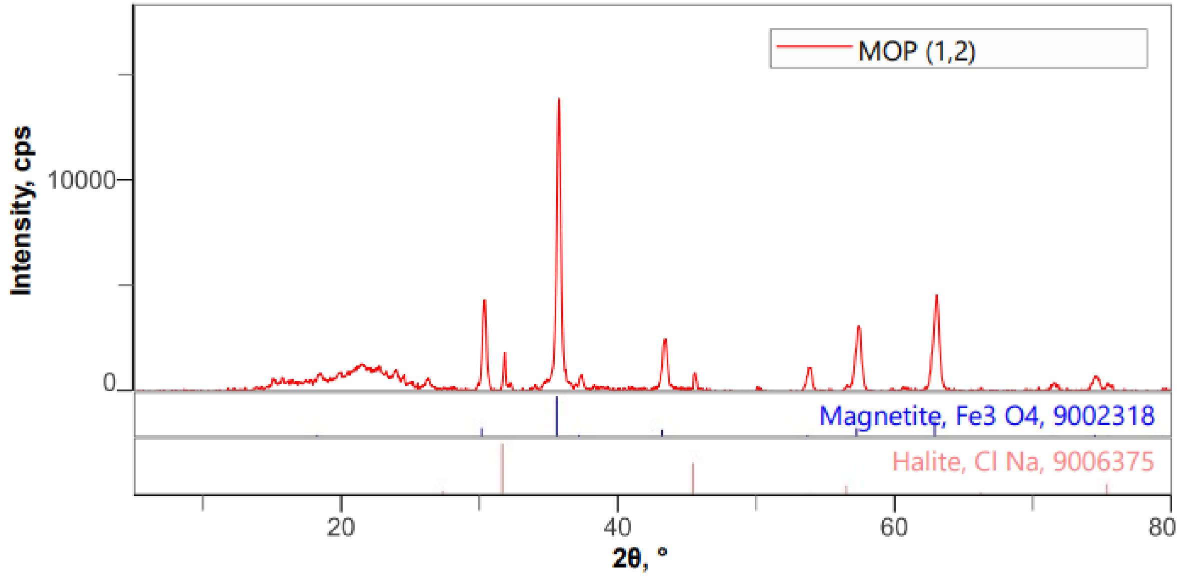

Figure S9 Qualitative analysis of MOP (1:2)

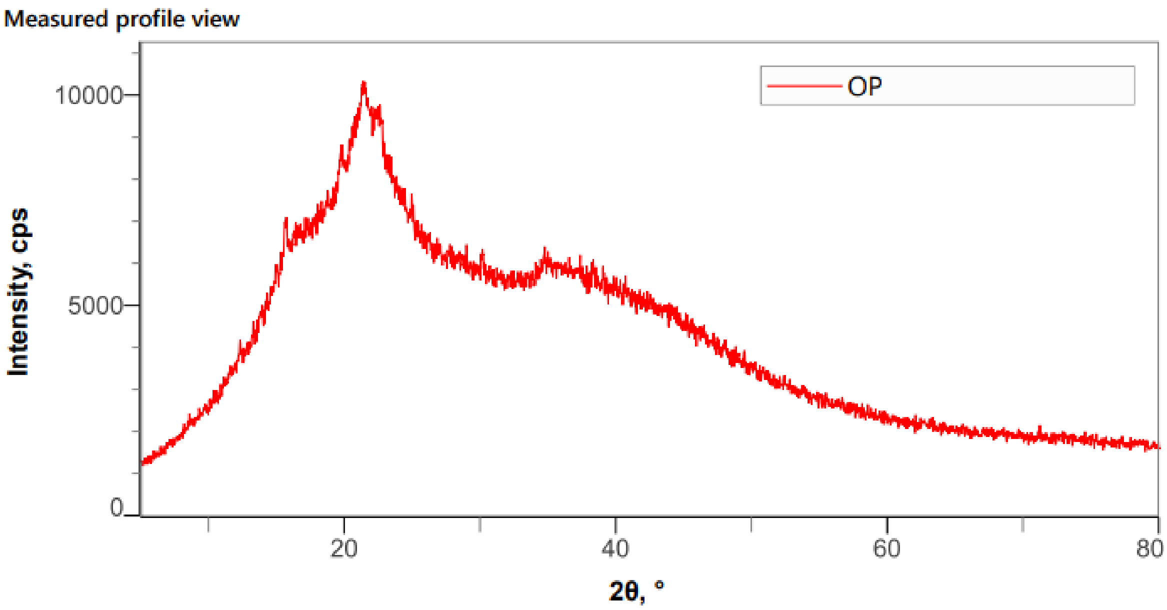

Figure S10 Qualitative analysis of OP

Qualitative Analysis Results

Phase name	Chemical formula	FOM	Phase reg. detail	Space Group	DB Card Number
Halite	Cl Na	1.284	S/M:COD	225 : Fm-3m	9006375
Graphite	C	2.380	S/M:COD	186 : P63mc	9008569
Magnetite	Fe3 O4	0.409	S/M:COD	227 : Fd-3m:2	9002318

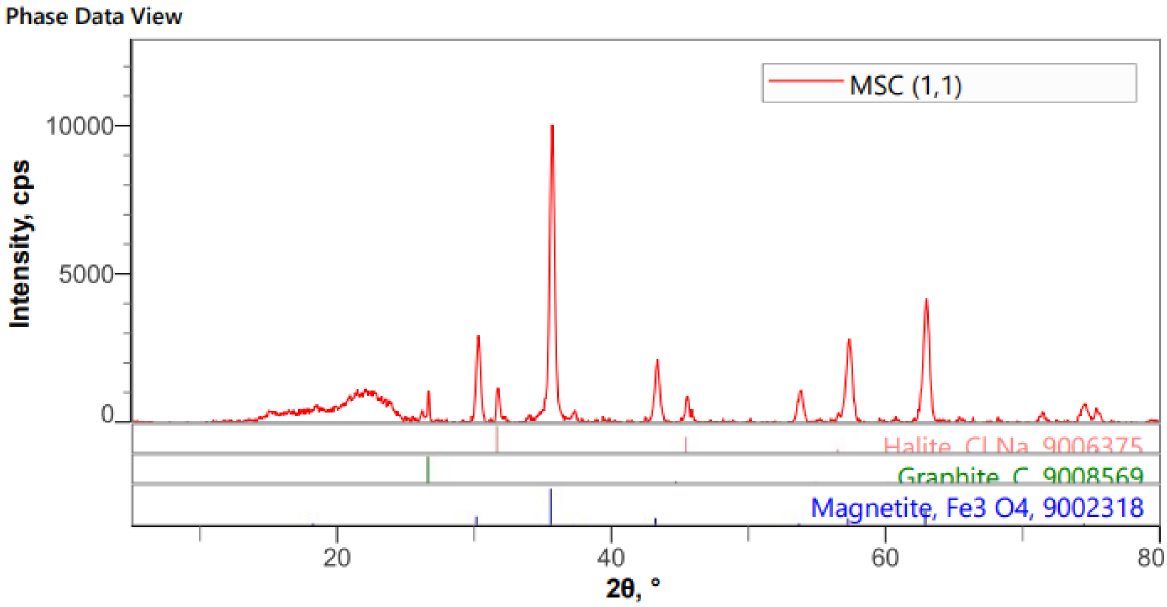

Figure S11 Qualitative analysis of MSC (1:1)

Qualitative Analysis Results

Phase name	Chemical formula	FOM	Phase reg. detail	Space Group	DB Card Number
Magnetite	Fe3 O4	0.409	S/M:COD	227 : Fd-3m:2	9002318
Halite	Cl Na	1.284	S/M:COD	225 : Fm-3m	9006375
Graphite	C	2.380	S/M:COD	186 : P63mc	9008569

Phase Data View

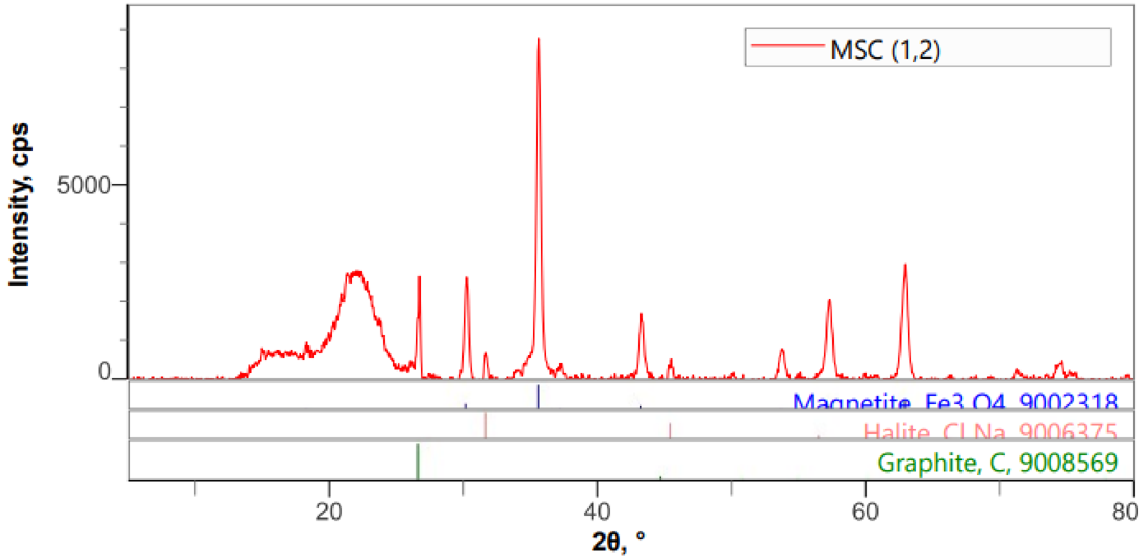

Figure S12 Qualitative analysis of MSC (1:2)

Qualitative Analysis Results

Phase name	Chemical formula	FOM	Phase reg. detail	Space Group	DB Card Number
Halite	Cl Na	1.310	S/M:COD	225 : Fm-3m	9006375
Magnetite	Fe3 O4	0.692	S/M:COD	227 : Fd-3m:2	9002318

Phase Data View

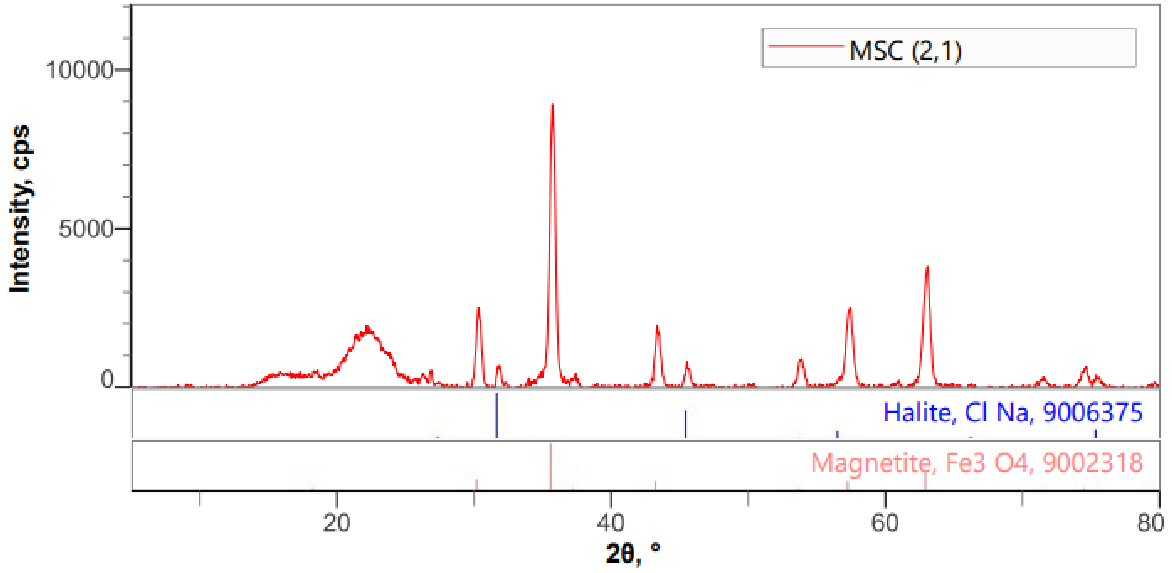

Figure S13 Qualitative analysis of MSC (2:1)
